# Prevalence and associated factors of goiter among rural children aged 6-12 years old in Northwest Ethiopia, cross -sectional study

**DOI:** 10.1186/1471-2458-14-130

**Published:** 2014-02-07

**Authors:** Molla Mesele, Getu Degu, Haimanot Gebrehiwot

**Affiliations:** 1 Department of Nutrition, Institute of Public Health, University of Gondar, Gondar, Ethiopia; 2 Department of Biostatistics and Epidemiology, Institute of Public Health, University of Gondar, Gondar, Ethiopia; 3 Department of Environmental and Occupational Health, Institute of Public Health, University of Gondar, Gondar, Ethiopia

**Keywords:** Goiter, Iodine deficiency, Children, Ethiopia

## Abstract

**Background:**

Goiter, an indicator of chronic iodine deficiency, is a major public health problem for populations living with iodine deficient environment, particularly for young children. It is a threat to the social and economic development of many developing countries including Ethiopia. The aim of the study was to assess the prevalence and associated factors of goiter among rural children aged 6-12 years, Northwest Ethiopia.

**Methods:**

A community based cross-sectional study was employed from July to December 2012 in Lay Armachiho district. A total of 698 children aged 6-12 years were included in the study. Multistage sampling was used. Children were examined for the presence/absence of goiter using a criterion set by World Health Organization. The level of Iodine of the salt was estimated by using spot testing kits. Descriptive and summary statistics were employed. Bivariate and multivariate logistic regressions were used to identify associated factors. The degree of association was assessed by using Odds ratio with 95% confidence interval were computed to see the presence and strength of association.

**Results:**

Totally 694 children were included in the analysis. The prevalence of goiter was found to be 37.6%. Goiter of grade 1 was 28.5% and that of grade 2 was 9.1%. 29.7% of the samples had adequate iodine content. The age of child (AOR: 1.24,95% CI: 1.12, 1.36), being female (A*OR =* 1.98, 95% CI: 1.38-2.85), salt iodine level (AOR = 0.44, 95% CI: 0.27, 0.71), family history of goiter (AOR = 3.18, 95% CI: 2.08, 4.858), fish consumption (AOR = 0.42, 95% CI; 0.22, 0.80) were factors associated with goiter.

**Conclusion:**

Chronic iodine deficiency was a severe public health problem in the study communities. Ensuring the consumption of iodized salt and promotion of fish intake at the household level are highly recommended.

## Background

Thyroid disorders are one of the most common endocrine problems in children and adolescents. Diagnostic approaches of thyroid disorders can be approached from the perspective of goiter [1]. The involvement of both dietary non dietary factors besides iodine deficiency might interact in the genesis of thyroid enlargement [2].

Goiter, an indicator of iodine deficiency, is a major public health problem for populations living in iodine deficient environment. Iodine deficiency during childhood would reduce somatic growth, cognitive and motor function [3,4].

Iodine deficiency is characterized by a slowdown of metabolic processes that results in growth and development abnormality for children [5,6]. It has multiple adverse effects in humans, termed as an iodine deficiency disorders (IDDs). This problem is happened due to inadequate production of thyroid hormone [4]. Iodine deficiency is the most common cause of preventable mental retardation. Subsequently, it would pose a threat to the social and economic development of countries [7].

Controlled studies conducted in iodine-deficient regions have shown that iodine supplementation eliminated new cases of cretinism. Furthermore, it contributed for the reduction of infant mortality and the improvement of cognitive function in the population [6].

School-age children are considered as an appropriate target group for determining iodine deficiency due to their susceptibility to iodine deficiency, easily accessibility as a study group and representativeness of their community society as a whole [8].

In the last two decades, the elimination of IDD has been considered as an integral part of many national nutrition strategies [9]. As a result, carefully monitored universal salt iodization program is the most effective strategy to control iodine deficiency. Achieving optimal iodine intake from iodized salt would minimize the amount of thyroid dysfunction in population [4,10].

Globally, the Total Goiter Prevalence (TGP) in the general population is estimated to be 15.8%, varying between 4.7% in America to 28.3% in Africa [3]. Prevalence of goiter in children aged 6–12 years varies in different studies in the world. It was 22.3% in southern Sudan, 11.4% Rajasthan, and 20.5% in India [11-13].

Study on prevalence of goiter among children 6 to 12 years of age in Ethiopia by the year 2005 was 39.9%, with rates of 27.7% for palpable, and 12.2% for visible goiter [14].

The prevalence of goiter was higher in females than males in different studies in the world [13,15,16]. Low dietary supply of iodine in areas where the soil has low iodine content is the main factor for development of Goiter. Fortification of salt with iodine is best method of preventing iodine deficiency in such kind of population [3,17,18].

According to the demographic and health survey report of 2011 only 16 percent of children in Ethiopia live in households that utilized an iodised salt. The percentage is higher in urban areas than in rural areas (24% compared with 14%) [17].

Environmental goitrogens may cause goiter by acting directly on the thyroid gland, but they can also act indirectly by altering the regulatory mechanisms of the thyroid gland and the peripheral metabolism and excretion of thyroid hormones. Different studies suggested that intake of goitrogens in certain food items like millet, cow milk, cassava, cabbage, sorghum and water can cause goiter [11,19,20].

Clinical examination of thyroid gland using the standard method which is one of the many approaches recommended by the joint WHO/UNICEF/ICCIDD consultation to identify IDD was used in the study. The other quantifiable indicators like UIE method and TSH were not considered in the study because they are costly and in accessible in poor settings, like Ethiopia.

IDD is more confined to mountainous regions were soil erosion and flooding is common. Lay Armachiho district is one of the mountainous areas in Ethiopia. This study assessed the prevalence of goiter and its associated factors among children of Lay Armachiho District.

## Methods

A community based cross-sectional study design was employed in Lay Armachiho district, northwestern Ethiopia from July to December 2012.

Those rural children aged 6–12 in the selected kebeles in Lay Armachiho district were included in the study. Those children who were seriously ill at the time of study were excluded from the study.

Household was considered as a sampling unit. Children with age group 6–12 years who lived in the study area along with their mothers/caregivers were the study units. Sample size was calculated using single population proportion formula by assuming the prevalence of goiter in children aged 6–12 years 29.1% [21], with 95% confidence interval, 5% absolute precision, 10% non response rate, and design effect of two, the total sample size was calculated to be 698.

Multi stage sampling was employed. The first stage was kebele (the smallest administrative unit in Ethiopia), whereas household was second stage. Among 33 rural *kebeles* in the district, seven of them were selected by simple random sampling. In each kebele a number of households with probability proportional to size were allocated. Then systematic random sampling technique was employed to reach individual households. The first houses were identified from the central places which were kebele administrative offices. By rotating a pencil on a book, where the sharp end indicating was taken to be the first household. Data collectors counted the total number of houses between the kebele administrative offices and the boundary of the kebeles. The sampling interval was calculated. The subsequent households were selected by using the sampling intervals. Data collectors were continuing their interview until the calculated samples were obtained.

In this particular study, the presence of goiter was considered absent if no palpable or visible goiter (Grade 0), whereas goiter is present when the child has Grade 1 or grade 2 goiter or both. Iodine level of salt was adequate when its level was 15 PPM or more.

A pre-tested and structured questionnaire was used. Physical examination of the thyroid was done to identify the presence of goiter. Iodine level of the salt was determined by a rapid test kit. The test can be used semi -quantitatively to measure iodine in salt at 0, 15, and ≥15 ppm depending on the intensity of the color obtained.

Six Health Officer supervised by 3 supervisors collected the data. Three days training was given before data collection. Pre-test of the instrument and the procedure was conducted on 34 households before the actual data collection. Physical examination of thyroid was performed by trained health officers and goiter was graded by using WHO grading system (Grade 0: no goiter, grade 1: palpable but not visible and Grade 2: visible). Salt at household level was measured by rapid MBI test kit. The data collector delivered one or two drops of the solution on a small salt sample (one teaspoon is adequate). The intensity of the blue color which develops indicates the salt iodine level.

First code was given to the completed questionnaire and then data were entered into EPI info version 3.5.3 statistical software and then transferred to SPSS version 20 statistical package for further analysis. Data cleaning were performed to check for accuracy and consistencies and missed values and variables. The results were presented in the form of tables, figures, and text. Both bivariate and multivariate logistic regressions were used to identify associated factors. Odds ratio with 95% confidence interval were used to identify the presence and strength of association.

Ethical clearance was obtained from the Institutional Ethical Review board of Institute of Public Health, College of Medicine and Health Sciences, University of Gondar. Written letters were offered from North Gondar Zonal Health Department. Verbal Informed consent was obtained from each mothers/caregivers before the start of the interview. Children were also asked for assent before palpation of their thyroid gland. Those children who were identified as having goiter were referred to health institutions to get appropriate treatment and support.

## Results and discussion

### Socio demographic characteristics

Out of 698 children, 694 children were included in the analysis (response rate = 99.42%). The mean age of the study participants’ was 8.6 years (SD = 1.93). Three hundred twenty three (46.5%) children were males. Majority (93.2%) of mothers/caregivers were Orthodox Christians. Five hundred fifty nine (80.5%) of mothers/caregivers were unable to read and write and 474(68.3%) were house wives (Table 1).

**Table 1:** Socio-demographic characteristics of children and their mothers/caregivers at rural kebeles of Lay Armachiho district, Northwest Ethiopia, 2012 (N = 694)

**Variables**	**Numbers**	**Percentage**
Sex of children		
Female	323	46.5
Male	371	53.5
Marital status of mothers/caregivers		
Single	20	2.9
Married	569	82.0
Divorced	70	10.1
Widowed	35	5.0
Ethnicity		
Amhara	337	48.6
Tigre	6	0.9
Kimant	351	50.6
Religion		
Orthodox	647	93.2
Muslim	41	5.9
Other*	6	0.9
Educational status of mother/caregiver		
Unable to read write	559	80.5
Able to read write	50	7.2
Primary education	65	9.4
Secondary education	14	2.0
Tertiary and above	6	0.9
Occupation of mother/caregiver		
Housewife	474	68.3
Farmer	96	13.8
Government employed	15	2.2
Merchant	75	10.8
Day laborer	31	4.5
Private employed	3	0.4
Kebele		
Workmidir	72	10.4
Worangeb/Maura	55	7.9
Janicaw	89	12.8
Woynochrobit	134	19.3
Genbera	110	15.9
Chiranbezo	132	19.0
Aykuachakirn	102	14.7

*Protestant, Catholic.

This study showed that five hundred forty seven (78.8%) of mothers/caregivers usually add salt late at the end of cooking. Most (87.5%) of the mothers/caregivers did not expose salt to sunlight while 587 (84.6%) of them covered the salt containers. Among household salt samples, iodine concentration varied from 0 ppm to 30 ppm by using MBI rapid test kits. Two hundred six (29.7%) households used salt with adequate iodine level (≥15 ppm) (Table 2).

**Table 2:** Knowledge and attitude of mothers on iodized salt and utilization of iodized salt in rural households, Lay Armachiho District, Northwest Ethiopia, 2012 (N = 694)

**Frequency**		**Percent**
Type of salt used		
Packed*	28	4.22
Course (not packed)	666	95.78
Time to add salt while cooking food		
Early	33	4.8
Middle	95	13.7
Late/end	547	78.8
After cooking	14	2.0
Don’t know/not sure	5	0.7
Sun light exposure		
Yes	82	11.8
No	607	87.5
How it stored (storage material)		
Kept open (open container)	101	14.6
Kept closed (closed container)	587	84.6
Both	5	0.8
Storage with relation to fire		
Near to fire	317	45.7
Away from fire	372	53.6
Both	5	0.7
Salt iodine level		
0 ppm	260	37.5
1-15 ppm	228	32.8
16-30 ppm	206	29.7

*Powdered salt in packaged material like plastic.

Millet and cabbage were goitrogens frequently eaten by children. Fish was also consumed by small number of children. Pond was their source of drinking water for majority (65.9%) of participants (Table 3).

**Table 3:** Dietary intake of children and consumption of goitrogens in rural children aged 6–12 in Lay Armachiho District, Northwest Ethiopia, 2012 (N = 694)

	**Frequency**	**Percent**
Millet consumption		
At least once per day	165	23.8
At least once per week	230	33.1
At least once per month	189	27.2
Never	110	15.6
Dairy product consumption		
At least Once per day	128	18.4
At least once per week	206	29.7
At least once per month	102	14.7
Never	258	37.2
Cabbage consumption		
At least once per day	42	6.3
At least once per week	216	30.9
At least once per month	198	28.5
Never	238	34.3
Fish consumption		
At least once per week	44	6.34
At least once per month	108	15.56
Never	542	78.09
Source of drinking water		
Pipe	134	19.3
Bare hole	70	10.1
River	33	4.8
Pond	457	65.9

### Prevalence of goiter

The overall prevalence of goiter was found to be 37.6%. Prevalence of Grade 1 goiter was 28.5% and that of grade 2 was 9.1%. In terms of the WHO, UNICEF and ICCIDD criteria for assessing the severity of IDD using prevalence of goiter, the prevalence in the study area could be seen as high and IDD was severe public health problem [8].

This finding was in agreement with findings of a national epidemiological goiter survey among children in Ethiopia which was 39.9% (95%CI: 38.6%, 41.2%) [14] and in Sudan (38.8%) [19].

The Prevalence in the current study was higher than in studies done at South Africa (25.5%) [22], southern Blue Nile area of Sudan (22.3%) [11], India (20.5%) [12], Rajasthan (11.4%) [13] but it was lower than a study done on schoolchildren in Islamabad which was 71.6% [23]. The Prevalence was also lower than the cross-sectional study done among ten villages from four administrative regions of Ethiopia with a gross prevalence of goiter among school children of 53.3% [20]. The reason may be due to the fact that our study was undertaken after universal salt iodization launched in Ethiopia.

The study revealed that prevalence of goiter was 98% higher among females when compared to that of males (A*OR =* 1.98, 95% CI: 1.38-2.85). This may be due to the fact that iodine requirement for female children were higher than males especially at the beginning of pubertal age. This is related to the difference in sex hormones and pubertal growth pattern among boys and girls in higher age groups. This finding was similar with different studies [13,15,16,20,21] but in a study conducted at Sudan and china there were no significant difference between boys and girls [11,14,24].

### Factors associated with goiter

For a unit increase in age, there was a 1.24 times increased risk of developing goiter in children (AOR: 1.24,95% CI: 1.12, 1.36) (Table 4). This might be due to the fact that iodine requirement increases with age. Older children had prolonged exposure for iodine deficient environment and also universal salt iodization was implemented in the past few years and older ones are less benefited from the intervention. Thyroid size is correlated with body surface area and increases with age. Hence, enlarged thyroid could be more visible and palpable in elderly children. This finding was similar with study done in Lesotho [19] but it was different from in china which indicated no statistically significant difference among the goiter rates across the different age groups [24].

**Table 4:** Factors associated with goiter among rural children aged 6–12 in Lay Armachiho District, Northwest Ethiopia, 2012 (N = 694)

	**OR (95% CI)**			
**Variables**	**Goiter status**		**Crude OR (95% CI)**	**Adjusted OR (95% CI)**
	Yes	No		
Age				
	261	433	1.208(1.113,1.310)	1.236(1.123,1.360)
Sex of the child				
Male	93	230	1	1*
Female	168	203	2.047(1.4922.807)	1.986 (1.379,2.860)
Type of salt used				
Packed	5	23	1	1
Course (not packed)	256	410	2.872(1.078,7.650)	2.708(0.868, 8.452)
Time to add salt during cooking				
Early	13	25	1	**
Middle	23	72	0.614(0.271,1.392	
Late end	224	323	1.334(0.668,2.663)	
After cooking	1	13	0.148(0.017,1.259)	
Storage with relation to fire				
Near to fire	139	183	1	**
Away from fire	122	250	0.642(0.472,0.875)	
Place of storage of salt				
Kept open	52	54	1.746(1.151,2.649)	1.625(0.985,2.680)
Kept closed	209	379	1	1
Family history of goiter				
Yes	125	93	3.360(2.406,4.693)	3.175(2.075,4.858)
No	136	340	1	1*
Kebele where the child live				
Workmidir	49	23	2.493(1.328,4.681)	2.831(1.237,6.479)
Worangeb/maura	6	49	0.143(0.056,0.364)	0.303(0.095,0.970)
Janicaw	24	65	0.432(0.235,0.794)	0.565(0.260,1.230)
Woynochrobit	62	72	1.008(0.601,1.689)	0.701(0.285,1.726)
Genbera	29	81	0.419(0.236,0.745)	0.454(0.207,0.997)
Chiranbezo	44	88	0.585(0.344,0.996)	1.704(0.602,4.822)
Aykochakirn	47	55	1	1*
Altitude in meter				
<1500 m	96	78	2.462(1.54,3.936)	**
1500-2000 m	53	146	0.726(0.450,1.173)	
2000-2500 m	68	121	1.124(0.704,1.795)	
>2500 m	44	88	1	
Knowledge on iodised salt				
Not knowledgeable	181	258	1	**
Knowledgeable	80	175	0.652(0.470,0.902)	
Attitude on iodised salt use				
Favorable	188	277	1	**
Not favorable	73	156	0.689(0.494,0.963)	
Millet consumption				
At least once per day	79	86	1	**
At least once per week	60	170	0.384(0.251,0.587)	
At least once per month	82	107	0.834(0.548,1.269)	
Never	40	70	0.622(0.380,1.020)	
Fish consumption				
At least once per week	5	39	0.214(0.083,0.552)	0.400(0.131,1.224)
At least once per month	53	55	1.609(1.062,2.438)	0.416(0.216,0.804)
Never	203	339	1	1*
Cereals commonly used by the child				
Millet	85	93	1	1
Sorghum	105	151	0.761(0.517,1.119)	0.481(0.266,0.869)
Teff	15	35	0.469(0.239,0.919)	0.596(0.276,1.289)
Maize	15	44	0.373(0.194,0.719)	0.508(0.226,1.140)
Wheat	39	106	0.403(0.252,0.644)	0.315(0.130,0.765)
Oat	2	4	0.547(0.98,3.063)	0.538(0.069,4.173)
Iodine level of salt used by household				
0 ppm	134	126	1	1*
1-15 ppm	74	154	0.452(0.312,0.653)	0.634(0.403,0.996)
16-30 ppm	53	153	0.326(0.219,0.484)	0.441(0.272,0.714)

*Significant from the multivariate logistic regression (Backward LR method).

**Not significant and fail to appear into the final multivariate logistic regression model.

Family history of goiter was significantly associated with goiter in this study. Children having goiter in first degree relative were 3.18 times more likely to develop goiter when compared with those who had no (AOR = 3.18,95% CI: 2.08,4.86) (Table 4). This finding was in agreement with a survey of goiter in Brazil [25] and Ethiopia [26]. In a case control study in Germany, Patients with goiter had showed a significantly higher proportion of parents or siblings with goiter. Children from parents’ with goiter showed a 2.7 fold increased risk of developing goiter [27]. Thus, the significantly higher rate of positive family histories of goiter in children indicates the importance of genetic factors in goiter development.

In this study the kebele where the child was living had independently associated with the development of goiter. Children who live in Workmidir kebele were 2.83 times more likely to have goiter than their counterparts in Aykuachakirn kebele (AOR = 2.83, 95% CI: 1.24, 6.48) where as children in Genbera and Worangeb kebele were 54.6% and 69.7% less likely to have goiter when compared with their counterparts in Aykuachakirn respectively ((AOR = 0.454, 95% CI: 0.207, 0.997), (AOR = 0.30, 95% CI: 0.095, 0.97)) (Table 4). Households using adequate salt iodine level in Workmidir and Aykuachakirn were low. Consumption of salt with inadequate iodine level may be the reason for increased risk of goiter in children of Aykuachakirn and Workmidir. Moreover increased consumption of fish by children in Genbera may contribute for decreased prevalence in children of this kebele.

Low dietary intakes of iodine contribute a lot for development of goiter. Hence Increasing dietary intake of iodine through consuming iodised salt is clearly the key towards eradication of goiter caused by iodine deficiency. Based on WHO recommendation salt iodine level should be adequate in 90% of households.

In Ethiopia efforts have been made to iodize the salt produced in Afdera and Dobi in Afar and Somali regions. The Federal Government of Ethiopia passed a mandatory salt regulation requiring all salt meant for human consumption to be iodized since March 2011 (33). Only 29.7% of households were using adequately iodised salt in the study area. This finding was lower than a studies done in India [12,16], But higher when compared with a national survey in Ethiopia (15%) and Jodhpur district of Rajasthan ( 18.5%) [13,17].

Utilization of iodised salt in the households was independently associated with development of goiter in children. Using adequately iodized salt (16–30 ppm) was protective for goiter by 55.9% than using non iodized salt (0 ppm) (AOR = 0.44, 95% CI: 0.27, 0.71). Using salt with Iodine level of 1–15 ppm was also protective for goiter by 36.6% while compared to using non iodised salt(0 ppm) (AOR = 0.63, 95% CI: 0.40, 0.99) (Table 4). This finding was similar with a study done on Qom city of Iran [20], But in a study on Isfaham city of Iraq there was no relationship between iodine intake and goiter [26].

In this study fish consumption at least once per month was 58.4% times protective for goiter than never taking fish at all (AOR = 0.42, 95% CI; 0.22, 0.80) (Table 4). Foods of marine origin have higher iodine content because marine plants and animals concentrate iodine from seawater. So sea foods including fish are naturally high in there iodine content so fish consumption will increase daily intake of iodine [4,28].

Many studies on prevalence of goiter in Ethiopia were school based cross sectional surveys whereas the current study was community based. This study tried to include children who might not been enrolled in school or might have dropped out of school due to social stigma created by IDD. The study also differs from studies done before because it showed chronic iodine deficiency and determine recent iodine intake. The study also revealed the existence of severe iodine deficiency in the study area after Ethiopia launched universal salt iodization. But the study failed to determine the causal relationships between goiter and the predictor variables due to its cross-sectional nature. Inter observer Bias both in grading of goiter and determination of salt iodine level by rapid test kit were also limitations of this study.

## Conclusion

Prevalence of goiter is found to be high and iodine deficiency disorder is a severe public health problem. In this study being female, elder age, family history of goiter, living in Aykuachakirn and Workmidir increases risk of having goiter in children. Fish consumption and adequate iodine level of salt were protective for goiter. Ensuring the consumption of iodized salt and promotion of fish intake at the household level are highly recommended.

## Consent

Written informed consent was obtained from the patient’s guardian/parent/next of kin for the publication of this report.

## Abbreviations

BCC: Behavioral change and communication; CSA: Central statistical agency of Ethiopia; DHS: Demographic and health survey; FAO: Food and Agricultural Organization; ICCIDD: International Council on Control of Iodine Deficiency Disorders; IDD: Iodine deficiency disorders; IEC: Information, Education and Communication; PPM: Parts per million; SNNPR: Southern Nation’s Nationalities and People Region; SPSS: Statistical package for social sciences; TGP: Total goiter prevalence; UNICEF: United Nation International Children’s Fund; WHO: World Health Organization.

## Competing interest

The authors declare that they have no competing interests.

## Authors’ contribution

MM was the corresponding author and made substantial contributions in conception and design, acquisition of data, analysis and interpretation of data and has been involved in drafting the manuscript. GD Participated in the design of the study and performed the statistical analysis. HG Participated in the design of the study, writing of results and discussion and has been involved in drafting the manuscript. All authors read and approved the final manuscript. MM wrote the paper.

## Pre-publication history

The pre-publication history for this paper can be accessed here:

http://www.biomedcentral.com/1471-2458/14/130/prepub
